# CRISPR-based screening pinpoints H2AZ1 as a driver of senescence in human mesenchymal stem cells

**DOI:** 10.1093/procel/pwae035

**Published:** 2024-06-19

**Authors:** Ming-Heng Li, Xiaoyu Jiang, Yaobin Jing, Kaowen Yan, Shi-Jia Bi, Si Wang, Shuai Ma, Guang-Hui Liu, Weiqi Zhang, Shuhui Sun, Jing Qu

**Affiliations:** State Key Laboratory of Stem Cell and Reproductive Biology, Institute of Zoology, Chinese Academy of Sciences, Beijing 100101, China; Key Laboratory of Organ Regeneration and Reconstruction, Institute of Zoology, Chinese Academy of Sciences, Beijing 100101, China; University of Chinese Academy of Sciences, Beijing 100049, China; Institute for Stem Cell and Regeneration, Chinese Academy of Sciences, Beijing 100101, China; Beijing Institute for Stem Cell and Regenerative Medicine, Beijing 100101, China; State Key Laboratory of Membrane Biology, Institute of Zoology, Chinese Academy of Sciences, Beijing 100101, China; Key Laboratory of Organ Regeneration and Reconstruction, Institute of Zoology, Chinese Academy of Sciences, Beijing 100101, China; University of Chinese Academy of Sciences, Beijing 100049, China; Institute for Stem Cell and Regeneration, Chinese Academy of Sciences, Beijing 100101, China; Beijing Institute for Stem Cell and Regenerative Medicine, Beijing 100101, China; State Key Laboratory of Membrane Biology, Institute of Zoology, Chinese Academy of Sciences, Beijing 100101, China; Key Laboratory of Organ Regeneration and Reconstruction, Institute of Zoology, Chinese Academy of Sciences, Beijing 100101, China; International Center for Aging and Cancer, Hainan Academy of Medical Sciences, Hainan Medical University, Haikou 571199, China; State Key Laboratory of Membrane Biology, Institute of Zoology, Chinese Academy of Sciences, Beijing 100101, China; Key Laboratory of Organ Regeneration and Reconstruction, Institute of Zoology, Chinese Academy of Sciences, Beijing 100101, China; University of Chinese Academy of Sciences, Beijing 100049, China; Institute for Stem Cell and Regeneration, Chinese Academy of Sciences, Beijing 100101, China; Beijing Institute for Stem Cell and Regenerative Medicine, Beijing 100101, China; State Key Laboratory of Stem Cell and Reproductive Biology, Institute of Zoology, Chinese Academy of Sciences, Beijing 100101, China; Key Laboratory of Organ Regeneration and Reconstruction, Institute of Zoology, Chinese Academy of Sciences, Beijing 100101, China; University of Chinese Academy of Sciences, Beijing 100049, China; Institute for Stem Cell and Regeneration, Chinese Academy of Sciences, Beijing 100101, China; Beijing Institute for Stem Cell and Regenerative Medicine, Beijing 100101, China; Advanced Innovation Center for Human Brain Protection and National Clinical Research Center for Geriatric Disorders, Xuanwu Hospital Capital Medical University, Beijing 100053, China; Aging Translational Medicine Center, International Center for Aging and Cancer, Beijing Municipal Geriatric Medical Research Center, Xuanwu Hospital, Capital Medical University, Beijing 100053, China; Aging Biomarker Consortium, Beijing 100101, China; State Key Laboratory of Membrane Biology, Institute of Zoology, Chinese Academy of Sciences, Beijing 100101, China; Key Laboratory of Organ Regeneration and Reconstruction, Institute of Zoology, Chinese Academy of Sciences, Beijing 100101, China; University of Chinese Academy of Sciences, Beijing 100049, China; Institute for Stem Cell and Regeneration, Chinese Academy of Sciences, Beijing 100101, China; Beijing Institute for Stem Cell and Regenerative Medicine, Beijing 100101, China; Aging Biomarker Consortium, Beijing 100101, China; State Key Laboratory of Membrane Biology, Institute of Zoology, Chinese Academy of Sciences, Beijing 100101, China; Key Laboratory of Organ Regeneration and Reconstruction, Institute of Zoology, Chinese Academy of Sciences, Beijing 100101, China; University of Chinese Academy of Sciences, Beijing 100049, China; Institute for Stem Cell and Regeneration, Chinese Academy of Sciences, Beijing 100101, China; Beijing Institute for Stem Cell and Regenerative Medicine, Beijing 100101, China; Advanced Innovation Center for Human Brain Protection and National Clinical Research Center for Geriatric Disorders, Xuanwu Hospital Capital Medical University, Beijing 100053, China; Aging Translational Medicine Center, International Center for Aging and Cancer, Beijing Municipal Geriatric Medical Research Center, Xuanwu Hospital, Capital Medical University, Beijing 100053, China; International Center for Aging and Cancer, Hainan Academy of Medical Sciences, Hainan Medical University, Haikou 571199, China; Aging Biomarker Consortium, Beijing 100101, China; CAS Key Laboratory of Genomic and Precision Medicine, Beijing Institute of Genomics, Chinese Academy of Sciences and China National Center for Bioinformation, Beijing 100101, China; University of Chinese Academy of Sciences, Beijing 100049, China; Institute for Stem Cell and Regeneration, Chinese Academy of Sciences, Beijing 100101, China; Aging Biomarker Consortium, Beijing 100101, China; State Key Laboratory of Membrane Biology, Institute of Zoology, Chinese Academy of Sciences, Beijing 100101, China; Key Laboratory of Organ Regeneration and Reconstruction, Institute of Zoology, Chinese Academy of Sciences, Beijing 100101, China; University of Chinese Academy of Sciences, Beijing 100049, China; Beijing Institute for Stem Cell and Regenerative Medicine, Beijing 100101, China; Beijing Anzhen Hospital, Capital Medical University, Beijing Institute of Heart Lung and Blood Vessel Diseases, Beijing 100029, China; State Key Laboratory of Stem Cell and Reproductive Biology, Institute of Zoology, Chinese Academy of Sciences, Beijing 100101, China; State Key Laboratory of Membrane Biology, Institute of Zoology, Chinese Academy of Sciences, Beijing 100101, China; Key Laboratory of Organ Regeneration and Reconstruction, Institute of Zoology, Chinese Academy of Sciences, Beijing 100101, China; University of Chinese Academy of Sciences, Beijing 100049, China; Institute for Stem Cell and Regeneration, Chinese Academy of Sciences, Beijing 100101, China; Beijing Institute for Stem Cell and Regenerative Medicine, Beijing 100101, China; Aging Biomarker Consortium, Beijing 100101, China; Beijing Anzhen Hospital, Capital Medical University, Beijing Institute of Heart Lung and Blood Vessel Diseases, Beijing 100029, China

## Dear Editor

Cellular senescence is characterized by growth arrest and the onset of a senescence-associated secretory phenotype (SASP) (Consortium et al., 2023; Liu et al., 2023; Zhang et al., 2023). Consequently, senescent cells that accumulate within aged organs have the capacity to disseminate pro-senescence and pro-inflammatory signals, promoting structural tissue deterioration and functional decline, culminating in organismal aging (Cai et al., 2022; Consortium et al., 2024). Stem cells, residing within tissues and endowed with remarkable abilities for self-renewal and differentiation, play pivotal roles in the repair and regeneration of injured or aged tissues. As individuals age, stem cells also undergo senescence and exhaustion, leading to a diminished capacity for tissue regeneration and repair. This decline is prominently associated with changes in the inherent flexibility of chromatin structure within stem cells, which instructs their differentiation into specific cell types. Consequently, extensive studies have highlighted the critical role of chromatin modifications in the context of stem cell senescence (Wu et al., 2024; Zheng et al., 2024). Hence, identifying the key regulators of chromatin structure holds great potential for alleviating stem cell senescence and organ aging (Bi et al., 2024; Liu et al., 2022).

The nucleosome constitutes the fundamental building block of eukaryotic chromatin. Each nucleosome consists of 146 bp of DNA wrapped around a core histone octamer. This octamer comprises two copies of each of the histone proteins H2A, H2B, H3, and H4. Additionally, the histone protein H1, enveloped by 20 bp of DNA, plays a role in connecting adjacent nucleosome cores and stabilizing the higher-order chromatin structure. Consequently, the dynamic remodeling of chromatin structure is governed by different histone proteins, and this regulation occurs primarily through a series of post-translational modifications on different histones. In addition to the canonical histones, a diverse number of histone variants that are expressed independently of DNA replication and persist throughout the cell cycle have been identified. These variants possess the capacity to substitute canonical histones and confer distinct chromatin structural properties, which in turn impact critical processes such as chromosome segregation, DNA repair, or transcription initiation (Martire and Banaszynski, 2020). However, our current knowledge regarding the interplay between histone variants and stem cell senescence remains limited. In this study, using CRISPR-mediated loss-of-function screening for histone variant-related genes (HVRGs), we identified that depletion of the H2A.Z variant histone 1 (*H2AZ1*) attenuates the senescence of human mesenchymal stem cells (hMSCs). Mechanistically, we demonstrated that H2AZ1 functions as a transcriptional repressor of pleiotrophin (PTN) by binding to enhancer regions, thereby promoting cellular senescence.

**Figure 1.**
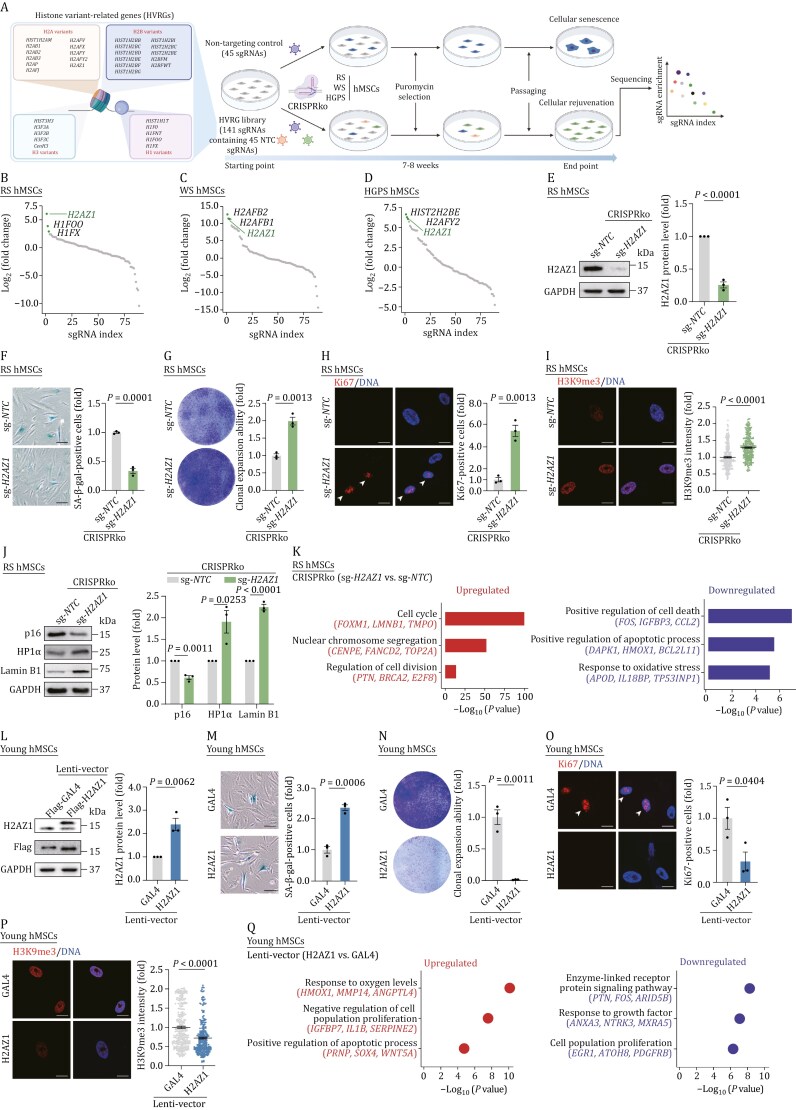
CRISPR-based screening identifies *H2AZ1* as a driver of cellular senescence. (A) Schematic of CRISPR-based loss-of-function (LOF) screening for histone variant-associated genes (HVRGs) to identify senescence drivers in RS, WS, and HGPS hMSCs. (B–D)Scatter plot showing the enriched sgRNAs of each gene in RS- (B), WS- (C), and HGPS- (D) hMSCs-based screening. The green dots indicate the top three hits. (E) Western blot analysis of H2AZ1 in RS hMSCs after CRISPR-mediated knockout (CRISPRko) of *H2AZ1*. GAPDH was used as the loading control. *n =* 3 biological replicates. Data are presented as the means ± SEMs. Two-tailed unpaired Student’s *t*-test was performed. (F–H) SA-β-gal staining (F), clonal expansion analysis (G), and immunofluorescence staining of Ki67 (H) in RS hMSCs after CRISPRko of *H2AZ1*. Scale bars, 100 μm (F) and 10 μm (H). *n =* 3 biological replicates. Data are presented as the means ± SEMs. Two-tailed unpaired Student’s *t*-test was performed. The white arrowheads in (H) indicate the Ki67-positive cells. (I) Immunofluorescence staining of H3K9me3 in RS hMSCs after CRISPRko of *H2AZ1*. Scale bars, 10 μm. *n* = 300 cells from three biological replicates. Data are presented as the means ± SEMs. Two-tailed unpaired Student’s *t*-test was performed. (J) Western blot analysis of the indicated proteins in RS hMSCs after CRISPRko of *H2AZ1*. GAPDH was used as the loading control. *n* = 3 biological replicates. Quantitative data (right) are presented as the means ± SEMs. Two-tailed unpaired Student’s *t*-test was performed. (K) Bar plot showing Gene Ontology (GO) terms and pathways enrichment analysis for upregulated (red, left) and downregulated (blue, right) differentially expressed genes (DEGs) in RS hMSCs after CRISPRko of *H2AZ1*. (L) Western blot analysis of H2AZ1 and Flag in young hMSCs transduced with lentiviruses expressing GAL4 or H2AZ1. GAPDH was used as the loading control. *n* = 3 biological replicates. Quantitative data (right) are presented as the means ± SEMs. Two-tailed unpaired Student’s *t*-test was performed. (M–O) SA-β-gal staining (M), clonal expansion analysis (N), and immunofluorescence staining of Ki67 (O) in young hMSCs transduced with lentiviruses expressing GAL4 or H2AZ1. Scale bars, 100 μm (M) and 10 μm (O). *n* = 3 biological replicates. Data are presented as the means ± SEMs. Two-tailed unpaired Student’s *t*-test was performed. The white arrowheads in (O) indicate the Ki67-positive cells. (P) Immunofluorescence staining of H3K9me3 in young hMSCs transduced with lentiviruses expressing GAL4 or H2AZ1. Data are presented as the means ± SEMs. Scale bars, 10 μm. *n* = 300 cells from three biological replicates. Data are presented as the means ± SEMs. Two-tailed unpaired Student’s *t*-test was performed. (Q) Dot plot showing GO terms and pathways enrichment analysis for upregulated (red, left) and downregulated (blue, right) DEGs in young hMSCs transduced with lentiviruses expressing H2AZ1 compared to GAL4.

To systematically explore the roles of histone variants in the regulation of hMSCs senescence, we constructed a CRISPR screening library targeting HVRGs. This library, denoted as HVRG library, comprises 96 single guide RNAs (sgRNAs) that target 5 H1 variants, 11 H2A variants, 11 H2B variants, and 5 H3 variants, with 3 sgRNAs assigned to each variant. Additionally, we included 45 non-targeting control sgRNAs (sg-*NTC*s) serving as controls (Figs. 1A and S1A–C). Subsequently, we conducted the CRISPR screening in three types of stem cell senescence models, including replicative senescent hMSCs (RS hMSCs) (Fig. S1D and S1E), Werner syndrome (WS, *WRN*-deficient) hMSCs (Fig. S1F and S1G), and Hutchinson-Gilford progeria syndrome (HGPS, carrying the heterozygous *LMNA*^*G608G*/+^ mutation) hMSCs (Fig. S1H and S1I). Notably, the latter two models represent established human stem cell models of premature aging (Wu et al., 2018). To ensure that the majority of cells harbor one sgRNA, we introduced the HVRG library at a low multiplicity of infection (MOI ≈ 0.3). In parallel, cells infected with lentivirus carrying sg-*NTC*s at the same MOI were utilized as control. After puromycin selection, we performed sequential cell passaging over an 8-week period until the cells infected with sgRNA carrying HVRGs had acquired a relatively rejuvenated phenotype in comparison to the control group, as determined by a reduced presence of senescent-associated β-galactosidase (SA-β-gal)-positive cells (Fig. S1J–L). These findings suggested that deficiency of certain histone variants could alleviate senescence in hMSCs. Subsequently, we harvested the rejuvenated cells and ranked the sgRNAs using DNA sequencing. *H2AZ1*, an H2A.Z histone variant, emerged as the only common hit in RS-, WS- and HGPS-based screening, the reduction of which retarded senescence in all three hMSCs senescence models (Fig. 1B–D).

To validate the rejuvenation effects of *H2AZ1* deficiency, we conducted lentivirus-mediated CRISPR knockout (CRISPRko) in senescent hMSCs (Fig. 1E). Relative to control cells, *H2AZ1* deletion did not impact the differentiation potential of hMSCs into osteoblasts, chondrocytes, and adipocytes (Fig. S1M–O) or genomic integrity (Fig. S1P). As anticipated, the ablation of *H2AZ1* ameliorated multiple senescent characteristics, as evidenced by a reduction in SA-β-gal-positive cells, restoration of compromised proliferation (e.g. enhanced clonal expansion, increased Ki67-positive cells, and EdU-positive cells) (Figs. 1F–H and S1Q), decreased expression of senescence marker p16 and SASP factors (e.g. *IL-6*, *CXCL8*), induction of Lamin B1, HP1α, H3K9me3, and lamina-associated polypeptide 2 (LAP2) expression (Figs. 1I, 1J, and S1R–T), decreased reactive oxygen species (ROS) level (Fig. S1U). Transcriptome sequencing (RNA-seq) revealed that *H2AZ1* deletion leads to upregulation of genes related to cell cycle and nuclear chromosome segregation, while genes linked to apoptosis and oxidative stress are downregulated (Figs. 1K, S1V, and S1W; Table S1). Additionally, in WS and HGPS hMSCs, the *H2AZ1* deficiency also attenuated cellular senescence (Fig. S2A–L), mirroring the effects observed in RS hMSCs. Moreover, in senescent models induced by ultraviolet (UV) irradiation, H_2_O_2_ treatment, or oncogene (H-Ras^V12^) transduction, *H2AZ1* depletion reduced the number of SA-β-gal-positive cells and improved their proliferation potential (Fig. S3A–L). Next, we assessed the pro-senescence effects of H2AZ1 by ectopic overexpression in wild-type hMSCs at early passage (young hMSCs) (Fig. 1L). As demonstrated by increased numbers of SA-β-gal-positive cells, impaired cell proliferation, and decreased expression of Lamin B1, HP1α, and H3K9me3, H2AZ1 overexpression accelerated senescence in young hMSCs (Fig. 1M–P and S4A). Moreover, H2AZ1 overexpression also suppressed the expression of genes associated with proliferation and responsiveness to growth factor, while inducing the expression of genes related to oxygen levels (Figs. 1Q, S4B and S4C; Table S1). In conclusion, these findings suggests that H2AZ1 plays a driving role in hMSCs senescence and that its depletion retards senescence across diverse biological contexts.

**Figure 2.**
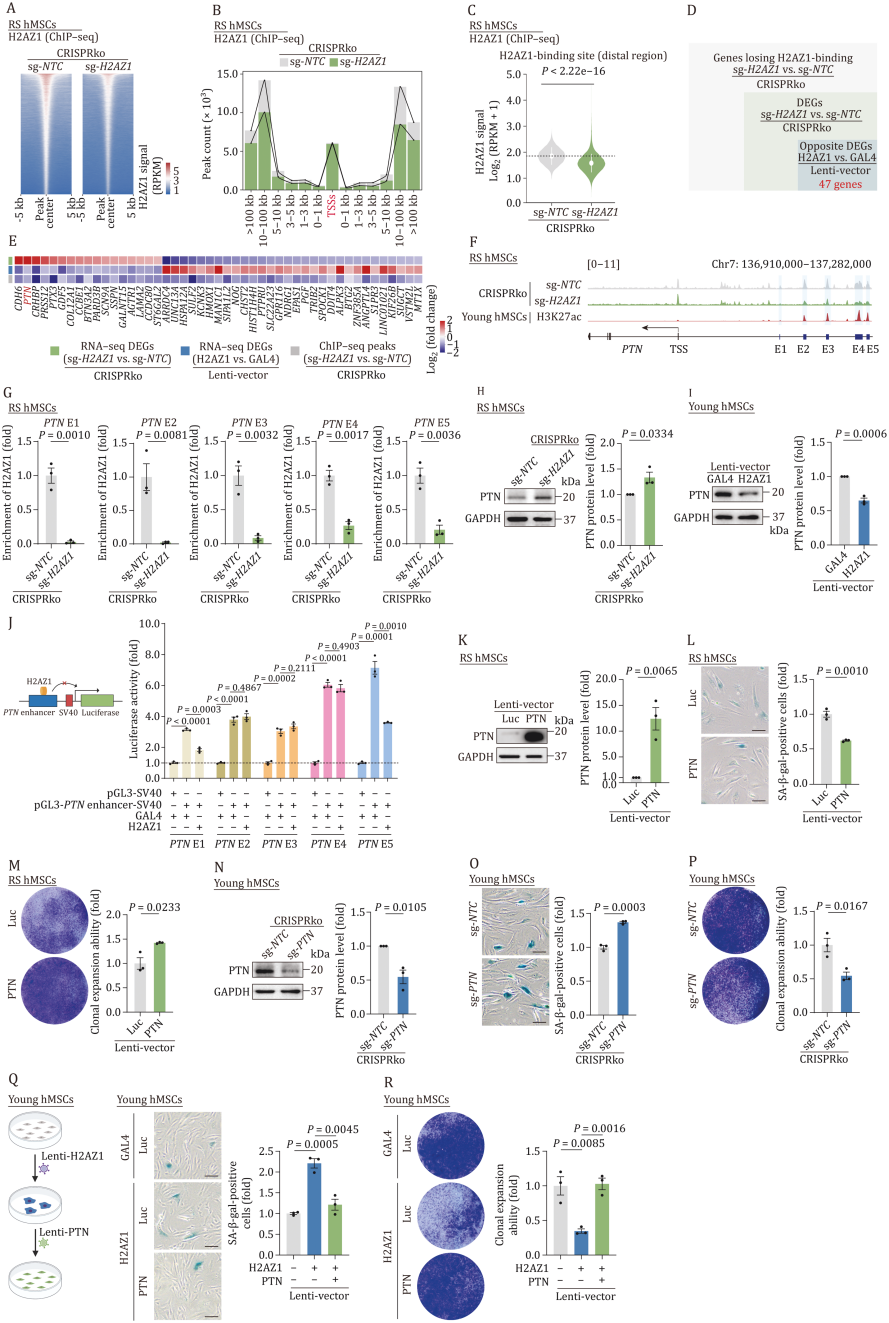
*H2AZ1* binds and modulates the *PTN* enhancer to promote hMSCs senescence. (A) Heatmap showing the H2AZ1 ChIP-seq signals in RS hMSCs after CRISPRko of *H2AZ1*. The color key from blue to red indicates signals (Reads Per Kilobase Million, RPKM) from low to high. (B) Bar plot showing the distance from H2AZ1-binding sites to TSSs in RS hMSCs after CRISPRko of *H2AZ1*. (C) Violin plot showing the H2AZ1 ChIP-seq signals at H2AZ1-binding distal regions (3 kb away from TSSs) in RS hMSCs after CRISPRko of *H2AZ1*. *P* value was calculated by a two-sided Wilcoxon signed-rank test. (D) Venn diagram showing the number (47) of genes regulated by H2AZ1 via binding at distal regions overlapping with DEGs caused by CRISPRko of *H2AZ1* in RS hMSCs but reversed by H2AZ1 overexpression in young hMSCs. (E) Heatmap showing H2AZ1-regulating genes that are differentially expressed in RS hMSCs by CRISPRko of *H2AZ1* but reversed in H2AZ1-transduced young hMSCs. The color key from blue to red indicates the Log_2_(fold change) of DEGs and the differential peak from low to high. (F) Snapshot showing the binding pattern of H2AZ1 on distal regions of *PTN* in *H2AZ1*-deficient RS hMSCs and the H3K27ac signals on distal regions of *PTN* in young hMSCs. H3K27ac ChIP-seq data were obtained from a previous study (Liu et al., 2022). The enhancers are marked with blue squares. (G) ChIP-qPCR analysis of the enrichment of H2AZ1 on the *PTN* enhancers in RS hMSCs after CRISPRko of *H2AZ1*. *n* = 3 biological replicates. Data are presented as the means ± SEMs. Two-tailed unpaired Student’s *t*-test was performed. (H and I) Western blot analysis of PTN in RS hMSCs after CRISPRko of *H2AZ1* (H) and young hMSCs transduced with lentiviruses expressing GAL4 or H2AZ1 (I). GAPDH was used as the loading control. *n =* 3 biological replicates. Data are presented as the means ± SEMs. Two-tailed unpaired Student’s *t*-test was performed. (J) Luciferase reporter assay measuring the enhancer activity of *PTN* E (1–5) by transfecting pGL3-*PTN* E (1–5)-luciferase and Renilla plasmids into H2AZ1-transduced hMSCs. The pGL3-promoter plasmid was used as a negative control. *n* = 3 biological replicates. Data are presented as the means ± SEMs. Two-tailed unpaired Student’s *t*-test was performed. (K, N) Western blot analysis of PTN in RS hMSCs transduced with lentiviruses expressing Luc or PTN (K) and young hMSCs after CRISPRko of *PTN* (N). GAPDH was used as the loading control. *n* = 3 biological replicates. Data are presented as the means ± SEMs. Two-tailed unpaired Student’s *t*-test was performed. (L and O) SA-β-gal staining of RS hMSCs transduced with lentiviruses expressing Luc or PTN (L), and young hMSCs after CRISPRko of *PTN* (O). Scale bars, 100 μm. *n =* 3 biological replicates. Data are presented as the means ± SEMs. Two-tailed unpaired Student’s *t*-test was performed. (M and P) Clonal expansion analysis of RS hMSCs (EP, early passage; LP, late passage) transduced with lentiviruses expressing Luc or PTN (M), and young hMSCs after CRISPRko of *PTN* (P). *n =* 3 biological replicates. Data are presented as the means ± SEMs. Two-tailed unpaired Student’s *t*-test was performed. (Q and R) SA-β-gal staining (Q) and Clonal expansion analysis (R) of H2AZ1-transduced young hMSCs after transduced with lentiviruses expressing PTN. Scale bars, 100 μm (Q). *n* = 3 biological replicates. Data are presented as the means ± SEMs. Two-tailed unpaired Student’s *t*-test was performed.

Given that H2AZ1 as an integrated nucleosome component likely modulates chromatin structure and subsequent gene expression, we performed chromatin immunoprecipitation (ChIP) followed by high-throughput sequencing to explore the potential genomic-binding regions of H2AZ1, aiming to uncover the mechanisms through which *H2AZ1* depletion alleviates senescence. Our analysis revealed a global reduction in H2AZ1 binding signals upon *H2AZ1* deficiency (Figs. 2A and S5A–E). In hMSCs, H2AZ1 was enriched at both transcriptional start sites (TSSs) and distal regions (Fig. S5F), suggesting that H2AZ1 may regulate senescence via its *trans*-activity in regulating gene expression. More specifically, the loss of H2AZ1-binding events predominantly occurred at distal regions, particularly those located at 3 kb away from TSSs, while its occupancy at TSSs was comparatively less affected (Fig. 2B and 2C). These data led us to speculate that H2AZ1 regulates gene expression by binding to their distal regulatory regions, which might be enhancer regions. Hence, we performed an integrated analysis of the ChIP-seq data and RNA-seq data, identifying 47 candidate genes that might be under the regulation of H2AZ1 through its *trans*-activity (Figs. 2D, 2E, and S5G). Among these genes, *PTN*, encoding pleiotrophin, a secreted growth factor that is essential for hippocampal neurogenesis (González-Castillo et al., 2015), was notably decreased in senescent hMSCs (Fig. S5H).

In the following experiments, we explored the relationship between H2AZ1 and PTN during hMSCs senescence. First, we validated that H2AZ1 occupancy was lost at the *PTN* enhancer regions in *H2AZ1*-deficient hMSCs by ChIP–qPCR analysis (Figs. 2F, 2G and S5I). We then found that PTN expression levels are upregulated in *H2AZ1*-deficient RS hMSCs and downregulated upon H2AZ1 overexpression in young hMSCs (Figs. 2H, 2I and S5J–L), suggesting that PTN is negatively correlated to H2AZ1 in hMSCs. Moreover, we found that H2AZ1 was enriched at five *PTN* enhancers (*PTN* E1–5) marked with H3K27ac (Fig. 2F) and that the occupancy of H3K27ac was enriched upon H2AZ1 deficiency (Fig. S5M). In addition, by assessing enhancer sequence activity (Table S2) using a luciferase reporter plasmid (pGL3-promoter vector), we found that all *PTN* enhancers showed increased luciferase signal. Conversely, H2AZ1 overexpression diminished the enhancer activity of *PTN* E1 and E5 (Fig. 2J), indicating that H2AZ1 exerts a *trans*-repressive effect on *PTN*.

To further elucidate the functional role of PTN in hMSCs senescence, we overexpressed PTN in senescent hMSCs and found a decreased percentage of SA-β-gal-positive cells (Fig. 2K–M), reminiscent of the effects observed upon H2AZ1 ablation. Conversely, the absence of PTN promoted hMSCs senescence (Fig. 2N–P), resembling the consequences of H2AZ1 overexpression. Importantly, the H2AZ1-induced senescent traits were ameliorated by PTN overexpression (Fig. 2Q and 2R). Collectively, these findings suggested that the suppression of PTN acts as a crucial downstream event induced by H2AZ1, reinforcing cellular senescence.

Here, we pioneered studies that identify the role of histone variants in the context of stem cell senescence. Our results revealed that the elimination of H2AZ1 alleviated hMSCs senescence by suppressing PTN expression. As a corroboration of our findings, PTN expression was reduced across a spectrum of aged cells and tissues, supported by data available in the Aging Atlas database. Given the crucial role of histone variants in maintaining chromatin structures, which are implicated in various physiological or pathological processes, the H2AZ1-PTN axis unveiled in this study could have implications beyond cellular senescence, warranting further exploration. It is worth noting that our findings are based on a loss-of-function screening platform, leaving room for the possibility that certain histone variants may play a geroprotective role, as opposed to the pro-senescence function of H2AZ1. Therefore, a gain-of-function screening approach may offer novel insights into the roles of histone variants during aging (Jing et al., 2023).

In alignment with previous reports showing enrichment of nucleosomes incorporating H2AZ1 at promoter regions (Wen et al., 2020), our study revealed enrichment of H2AZ1 at both enhancer and promoter regions across the genome. This study provides the initial evidence that H2AZ1, functioning as a distal regulatory factor, exerts *trans*-repressive control over PTN, consequently promoting senescence in hMSCs. H2AZ1 is well-recognized for its involvement in cell fate determination and cell cycle regulation by replacing canonical H2A within chromosomes, subsequently affecting chromatin structure and gene expression. Hence, beyond its distal *trans*-regulatory role, exploring whether H2AZ1 plays a role in reshaping chromatin high-order structures during cellular senescence represents an exciting topic for further investigation. Furthermore, the upregulation of H2AZ1 and downregulation of PTN were observed in the muscles of aged monkeys (Jing et al., 2022). These suggested that targeting H2AZ1-PTN axis might offer a promising approach to ameliorating senescence in various cell types and addressing age-related diseases.

In this study, we employed a CRISPR/Cas9-based screening approach to systematically investigate the role of histone variants in human stem cell senescence and uncovered H2AZ1 as a novel driver of hMSCs senescence. Our results not only advance our comprehension of the functions of histone variants in aging but also lay the groundwork for further explorations on H2AZ1 as a potential target for intervention in aging and aging-related diseases.

## Supplementary data

Supplementary data is available at https://doi.org/10.1093/procel/pwae035.

pwae035_suppl_Supplementary_Figures_S1-S5

pwae035_suppl_Supplementary_Table_S1

pwae035_suppl_Supplementary_Table_S2

pwae035_suppl_Supplementary_Table_S3

pwae035_suppl_Supplementary_Materials

## Footnotes

We are grateful to Lei Bai, Ruijun Bai, Qun Chu, Shangyi Qiao, Xiangmei Jin, Luyang Tian, Jing Lu, and Ying Yang for their administrative assistance. We thank Wei Wang for providing WS hMSCs and Lanzhu Li for providing HGPS hMSCs. We also thank the Biorender platform for schematic diagram.

The authors declare no competing interests and their agreement to publish. All of them confirm their willingness to participate. All institutional and national guidelines for the care were followed.

This work was supported by the National Natural Science Foundation of China (82125011, 81921006, 92149301, 82322025, 82122024), the National Key Research and Development Program of China (2020YFA0804000, 2022YFA1103700, 2020YFA0112200, 2021YFF1201000, the STI2030-Major Projects-2021ZD0202400), the National Natural Science Foundation of China (92168201, 82330044, 32341001, 92049304, 92049116, 32121001, 82192863, 82071588, 82361148130, 82361148131, 82271600), CAS Project for Young Scientists in Basic Research (YSBR-076, YSBR-012), the Program of the Beijing Natural Science Foundation (Z230011), the Informatization Plan of Chinese Academy of Sciences (CAS-WX2022SDC-XK14), New Cornerstone Science Foundation through the XPLORER PRIZE (2021-1045), Youth Innovation Promotion Association of CAS (E1CAZW0401, 2022083), Excellent Young Talents Program of Capital Medical University (12300927), The Project for Technology Development of Beijing-affiliated Medical Research Institutes (11000023T000002036310), Excellent Young Talents Training Program for the Construction of Beijing Municipal University Teacher Team (BPHR202203105), and Young Elite Scientists Sponsorship Program by CAST (2021QNRC001).

J.Q., S.S., W.Z., and G.-H.L. conceptualized the work and supervised overall experiments. M.-H.L. performed the cell culture, CRISPR screening, and phenotypic and mechanistic analyses. X.J. performed bioinformatics analyses. Y.J. performed plasmid construction, RT–qPCR. K.Y. performed luciferase reporter analysis. S.-J.B. performed ChIP-qPCR. S. W. performed immunofluorescence staining. S.M. helped with bioinformatics analyses. M.-H.L., X.J., and Y.J. performed data analysis. J.Q., S.S., W.Z., G.-H.L. M.-H.L., X.J., and Y.J. performed manuscript writing, review, and editing. All authors reviewed the manuscript.

All data associated with this study are present in the paper or the Supplementary Materials. The raw data of screening, WGS data, transcriptomic data, and ChIP-seq data obtained have been deposited in the Genome Sequence Archive in the National Genomics Data Center, Beijing Institute of Genomics (China National Center for Bioinformation) of the Chinese Academy of Sciences, under accession number HRA005568. The raw H3K27ac ChIP-seq data for WT hMSCs: HRA001144 (Liu et al., 2022).

## References

[CIT0001] Bi S, Jiang X, Ji Q et al The sirtuin-associated human senescence program converges on the activation of placenta-specific gene PAPPA. Dev Cell 2024;59:991–1009.e12. 38484732 10.1016/j.devcel.2024.02.008

[CIT0002] Cai Y, Ji Z, Wang S et al Genetic enhancement: an avenue to combat aging-related diseases. Life Med 2022;1:307–318. 39872744 10.1093/lifemedi/lnac054 PMC11749557

[CIT0003] Consortium AB, Bao H, Cao J et al Biomarkers of aging. Sci China Life Sci 2023;66:1–174. 37076725 10.1007/s11427-023-2305-0 PMC10115486

[CIT0004] Consortium AB, Jiang M, Zheng Z et al A biomarker framework for liver aging: the Aging Biomarker Consortium consensus statement. Life Med 2024;3:lnae004. 39872390 10.1093/lifemedi/lnae004 PMC11749002

[CIT0005] González-Castillo C, Ortuño-Sahagún D, Guzmán-Brambila C et al Pleiotrophin as a central nervous system neuromodulator, evidences from the hippocampus. Front Cell Neurosci 2015;8:443. 25620911 10.3389/fncel.2014.00443 PMC4287103

[CIT0007] Jing Y, Zuo Y, Yu Y et al Single-nucleus profiling unveils a geroprotective role of the FOXO3 in primate skeletal muscle aging. Protein Cell 2022;14:499–514. 10.1093/procel/pwac061 PMC10305740 36921027

[CIT0006] Jing Y, Jiang X, Ji Q et al Genome-wide CRISPR activation screening in senescent cells reveals SOX5 as a driver and therapeutic target of rejuvenation. Cell Stem Cell 2023;30:1452–1471.e10. e1410. 37832549 10.1016/j.stem.2023.09.007

[CIT0009] Liu Z, Ji Q, Ren J et al Large-scale chromatin reorganization reactivates placenta-specific genes that drive cellular aging. Dev Cell 2022;57:1347–1368.e12. 35613614 10.1016/j.devcel.2022.05.004

[CIT0008] Liu X, Jiao H, Zhang B et al Migrasomes trigger innate immune activation and mediate transmission of senescence signals across human cells. Life Med 2023;2:lnad050. 39872064 10.1093/lifemedi/lnad050 PMC11749555

[CIT0010] Martire S, Banaszynski LA. The roles of histone variants in fine-tuning chromatin organization and function. Nat Rev Mol Cell Biol 2020;21:522–541. 32665685 10.1038/s41580-020-0262-8 PMC8245300

[CIT0011] Wen Z, Zhang L, Ruan H et al Histone variant H2A. Z regulates nucleosome unwrapping and CTCF binding in mouse ES cells. Nucleic Acids Res 2020;48:5939–5952. 32392318 10.1093/nar/gkaa360 PMC7293034

[CIT0013] Wu Z, Zhang W, Song M et al Differential stem cell aging kinetics in Hutchinson–Gilford progeria syndrome and Werner syndrome. Protein Cell 2018;9:333–350. 29476423 10.1007/s13238-018-0517-8 PMC5876188

[CIT0012] Wu Z, Qu J, Zhang W et al Stress, epigenetics, and aging: unraveling the intricate crosstalk. Mol Cell 2024;84:34–54. 37963471 10.1016/j.molcel.2023.10.006

[CIT0014] Zhang B, Yan H, Liu X et al SenoIndex: S100A8/S100A9 as a novel aging biomarker. Life Med 2023;2:lnad022. 39872551 10.1093/lifemedi/lnad022 PMC11749476

[CIT0015] Zheng Z, Li J, Liu T et al DNA methylation clocks for estimating biological age in Chinese cohorts. Protein Cell 2024;15:575–593. 38482631 10.1093/procel/pwae011 PMC11259550

