# Supplementary material for: CRISPR-based screening pinpoints H2AZ1 as a driver of senescence in human mesenchymal stem cells
Source: Protein Cell. 2024 Jun 19;16(4):293–9. doi: 10.1093/procel/pwae035 (PMC12082294; doi:10.1093/procel/pwae035)
Supplement: pwae035_suppl_Supplementary_Materials [file pwae035_suppl_supplementary_materials.pdf]

Supplemental Materials

Materials and methods

Cell culture

hMSCs were cultured in culture medium comprising 90% α-Minimum Essential Medium with GlutaMAX (Gibco), 10% fetal bovine serum (FBS, Gibco, 42Q3194K), 1% penicillin/streptomycin (Gibco), 1% non-essential amino acids (Gibco) and 1 ng/mL fibroblast growth factor 2 (FGF2, Joint Protein Central). WT hMSCs exhibit growth arrest in late-passage (LP, P = 17), therefore, P17 hMSCs are defined as RS hMSCs. HGPS and WS hMSCs exhibit growth arrest at P8-9 as previously reported (Zhang et al., 2015; Wu et al., 2018), therefore, P8 of HGPS hMSCs and P9 of WS hMSCs were selected for experimental analysis.

HEK293T cells were cultured using culture medium consisting of 90% DMEM/High Glucose (HyClone), 10% FBS (Gibco, A33H00K), 1% GlutaMAX (Gibco), 1% penicillin/streptomycin (Gibco), and 1% non-essential amino acids (Gibco).

All cells were cultured in a humidified incubator with 5% CO_2 at 37°C.

Construction of CRISPR library and plasmids

sgRNAs were annealed and cloned into LentiCRISPRv2 plasmid (Addgene #52961) after digestion with Esp 3I (Thermo Fisher Scientific # FD0454). The sgRNA sequences targeting all histone variants were designed according to the human GeCKOv2 library (Addgene #1000000048), with each gene corresponding to three distinct sgRNAs. The sgRNA sequences for HVRG library are listed in Table S3.

For overexpression of H2AZ1 and PTN, coding sequences (CDS) were amplified from hMSC cDNA with HS DNA Polymerase (Takara), then cloned into pLE4-GFP vector digested with XhoI and MluI enzymes.

For detecting *PTN*-enhancer activities, the sequences of *PTN* enhancers were amplified from hMSC genomic DNA and cloned into pGL3-SV40-promoter vector digested with XhoI and MluI enzymes.

The oligo sequences used in this study are listed in Table S3.

Lentivirus production

HEK293T cells were co-transfected with psPAX2 (Addgene #12260), pDM2.G (Addgene #12259) and transfer plasmids using Lipofectamine 3000 transfection reagent (Thermo Fisher Scientific). After 8 hours (hr), fresh medium was replaced. The supernatant was collected at 24 hr and 48 hr after transfection, followed by concentration with 19,400 rpm at 4°C. Then the lentiviruses were resuspended by PBS and stored at -80°C.

CRISPR/Cas9 based LOF screening

The CRISPR/Cas9-based LOF screening was performed as described previously (Wang et al., 2021). In detail, 1.5 × 10^6 cells (3,000 x coverage) of RS, WS and HGPS hMSCs were infected with HVRG library virus at MOI 0.3. After 72 hr of infection, 1 μg/mL puromycin (InvivoGen) was utilized to select the sgRNA-transduced cells. Subsequently, cells underwent continuous passaging until control cells exhibited growth arrest (8 weeks for RS hMSCs; 7 weeks for WS and HGPS hMSCs). Then the rejuvenated cells in HVRG library group were collected, and the genomic DNA was isolated using the DNeasy Blood & Tissue Kit (QIAGEN) following the manufacturer’s instruction. Then the DNA libraries were constructed by two-step PCR amplifications as previous reported (Wang et al., 2021; Li et al., 2023). The Nova-PE150 was used for DNA sequencing according to the manufacturer’s instruction. The primers used in this study are listed in Table S3.

SA-β-gal staining

SA-β-gal staining was performed as previously described (Bi et al., 2020). In brief, cells were cultured at 80% confluence and fixed with fixation buffer consisting of 2% formaldehyde and 0.2% glutaraldehyde for 5 minutes (min) at room temperature (RT). Subsequently, the cells were incubated with staining buffer at 37°C overnight. The percentage of SA-β-gal-positive cells was quantified by image J (version 1.48).

Clonal expansion assay

The clonal expansion assay was performed as previously described (Li et al., 2020). In summary, 5,000 hMSCs were seeded into a 12-well plate coated with gelatin, followed by continued culture for two weeks. Subsequently, the cells were fixed with 4% paraformaldehyde solution (PFA) for 10 min and subjected to crystal violet staining (Frdbio BHC0520) for 1 hr at RT. The relative cell density was quantified by Image J (version 1.48).

Immunofluorescence staining

Immunofluorescence staining was performed as previously described (Liu et al., 2012). Cells cultured on coverslips at 70%~80% confluency were fixed with 4% PFA for 15 min at RT. Subsequently, the cells were permeabilized with 0.4% Triton X-100 for 10 min and blocked with 10% donkey serum for 1 hr at RT, followed by incubation with primary antibodies at 4°C overnight. The next day, the cells were incubated with secondary antibodies (Thermo Fisher Scientific) and Hoechst 33342 (Invitorgen) at RT for 1 hr. Imaging was performed using ZEISS LSM900 confocal microscope and the cellular intensity was quantified by Image J (version 1.48). The antibodies used in this study are listed in Table S3.

EdU staining

The detection of EdU-positive cells was conducted using the BeyoClick™

EdU-488 Cell Proliferation Assay Kit (C0071L). Cells cultured on coverslips at 70%~80% confluency were treated with 10 μM EdU for 3 hr. After removing the culture medium, the cells were fixed with 4% PFA for 15 min at RT. Subsequently, the cells are permeabilized with 0.4% Triton X-100 for 10 min. Next, adding 150 μL of Click reaction solution to each culture well, and incubate at RT in the dark for 30 min. After washing three times with PBS, the cell nuclei were stained with Hoechst 33342 at room temperature for 10 min in the dark. Imaging was performed using ZEISS LSM900 confocal microscope.

Reverse transcription-quantitative real time polymerase chain reaction (RT–qPCR)

Total RNA was extracted with TRIzol reagent (Invitrogen) following the manufacturer's instruction. Subsequently, 2 μg RNA were reverse-transcribed into complementary DNA (cDNA) via the GoScript Reverse Transcription System (Promega). Quantitative PCR was conducted employing iTaq Universal SYBR Green Supermix (Bio-Rad). The qPCR primers used in this study are listed in Table S3.

Western blot analysis

Cells were collected and lysed with SDS-lysis buffer and boiled at 105°C for 10 min. After that, BCA Kit (Thermo Fisher Scientific) was used to quantify the protein concentration. 20 μg protein was loaded into SDS-PAGE gel for electrophoresis and transferred into PVDF membranes (Millipore). Subsequently, 5% skim milk powder was used for blocking. Then, the membranes were incubated with primary antibodies at 4°C overnight, followed by incubation with corresponding HRP-conjugated secondary antibodies for 1 hr at RT. Images were taken using Image Lab software. The relative protein levels were quantified by Image J (version 1.48). The antibodies used in this study are listed in Table S3.

Measurement of reactive oxygen species (ROS)

Cellular ROS levels were quantified utilizing H2DCFDA staining (Invitrogen, #C6827). In brief, the cells were treated with H2DCFDA (2.5 μM) for 30 minutes at 37°C in dark, and then analyzed by flow cytometry (BD LSRFortessa).

ChIP–seq library preparation and ChIP–qPCR

ChIP was performed as previously reported (Zhao et al., 2022; Jing et al., 2023). Briefly, cells were cross-linked with 1% formaldehyde for 10 min at RT with continuous rotation and quenched with 125 mM/L Glycine for 5 min. After cells were lysed on ice for 10 min, chromatin was broken using Covaris S220. Then the Dynabeads Protein A (Thermo Fisher Scientific) pre-conjugated with antibodies were added and incubated overnight at 4°C. The next day, DNA fragments were reverse-crosslinked at 68°C and extracted with phenol-chloroform-isoamyl alcohol extraction. Then, ChIP–qPCR and ChIP–seq were performed. The antibodies and primers for ChIP–qPCR are listed in

Table S3.

ChIP–seq libraries were prepared using KAPA Hyper Prep Kits (KAPA Biosystems) and Next Multiplex Oligos for Illumina (Index Primers Set 1) (New England Biolabs) according to the manufacturer's instruction, and then sequenced on Ilumina NovaSeq 6000 platforms.

Enzyme-Linked Immunosorbent Assay (ELISA)

The IL-6 secretion of hMSCs was assessed by an ELISA kit (Biolegend) following the manufacturer's instructions. In brief, cell culture supernatants were collected and incubated in an anti-IL6 coated plate. Next, sequentially add Avidin-HRP reagent and TMB substrate, and mix well. Then stop solution should be added to wells to terminate the reaction. The optical density of the plate was measured at 450 nm by Synergy H1 Microplate Reader (Biotek). IL-6 concentrations were normalized to the corresponding cell numbers.

Next-generation sequencing data analysis of CRISPR-based screening

The CRISPR-based screening sequencing results were calculated using MAGeCK software (version 0.5.9.2) (Li et al., 2014). The "count" subcommand first trimmed and counted the data, followed with a statistical test on a specified count table using the "test" subcommand. The "count_spacers.py" script (Joung et al., 2017) was used to determine the quality control of the HVRG library.

Copy number variation (CNV) data analysis

The reads in the whole genome sequencing (WGS) data were initially filtered using Trim Galore (version 0.6.7) software. Using Bowtie2 (version 2.2.5) software (Langmead and Salzberg, 2012), the remaining reads were mapped to the hg19. Samtools (version 1.6) (Li et al., 2009) was then used to convert the sam file into a bam file. For each 500-kb bin size, ReadCounter in HMMcopy_utils was used to tally the reads. Analysis of CNV was done using the R tool HMMcopy.

RNA–seq data analysis

We utilized Trim Galore (version 0.6.7) to trim and check the quality of the RNA–seq data. Then, HISAT2 (version 2.2.1) (Kim et al., 2015) was used to map clean data to the hg19. The number of reads mapped to each gene was determined using featureCounts (version 2.0.1) (Liao et al., 2014). Differentially expressed genes (DEGs) were determined using DEseq2 (version 1.30.1) (Love et al., 2014) with the criteria of an adjusted *P* value less than 0.05 and $|\log_2$ (fold change)$|$ greater than 0.58. The enrichment of Gene Ontology (GO) terms and pathways was conducted by Metascape (Zhou et al., 2019). Data reproducibility was evaluated using Euclidean distance in the R environment. The DEGs are listed in Table S1.

ChIP–seq data analysis

Trim Galore (version 0.6.7) was used on H2AZ1 ChIP–seq data for quality control and adaptor removal. The remaining reads were aligned to hg19 using Bowtie2 (version 2.2.5). We gathered the same amount of reads for subsequent analysis (147,000,000 for H2AZ1 ChIP–seq) to reduce the offset. DeepTools (version 3.5.1) (Ramírez et al., 2016) was used to convert bam data to bw files, and deepTools was used to generate heatmap files or profile files before optimizing them using R. The peak's genomic elements and distance from the TSSs were annotated and assessed with the ChIPseeker package (version 1.26.2) (Yu et al., 2015). DiffBind (Ross-Innes et al., 2012) was used to calculate the differential peak. The track image was created with the pyGenomeTracks software (version 3.7).

Statistical analysis

Data are presented as the means ± SEMs. *P* value (*P*) was computed by two-tailed Student's t test using GraphPad Prism (version 9.0) software. *P* values are presented in figures and statistical methods are clarified in figure legends.

Supplemental Figure Legends

Figure S1. CRISPR-based screening identifies *H2AZ1* as a driver of cellular senescence.

(A) Quality control of HVRG library.

(B, C) Windrose plots showing the percentage of mapped reads (B) and the count of mapped reads per sgRNA (C) by CRISPR-based screening in RS, WS and HGPS hMSCs.

(D, E) SA-β-gal staining (D) and clonal expansion analysis (E) of RS hMSCs (EP; LP). Scale bars, 100 μm. *n* = 3 biological replicates. Data are presented as the means ± SEMs. Two-tailed unpaired Student's t test was performed.

(F) Western blot analysis of WRN in wild-type (WT) and WS hMSCs. β-Tubulin was used as the loading control.

(G) Clonal expansion analysis of WT and WS hMSCs. *n* = 3 biological replicates. Data are presented as the means ± SEMs. Two-tailed unpaired Student's t test was performed.

(H) Genotyping showing the mutation of $LMNA^{G608G/+}$ in WT and HGPS hMSCs.

(I) Clonal expansion analysis of WT and HGPS hMSCs. *n* = 3 biological replicates. Data are presented as the means ± SEMs. Two-tailed unpaired Student's t test was performed.

(J-L) SA-β-gal staining of RS (J), WS (K), and HGPS (L) hMSCs transduced with HVRG library and control at 8 weeks for RS hMSCs, and 7 weeks for WS, and HGPS. Scale bars, 100 μm. *n* = 3 biological replicates. Data are presented as the means ± SEMs. Two-tailed unpaired Student's t test was performed.

(M-O) Characterization of osteogenetic (M), chondrogenetic (N) and adipogenetic (O) potential of RS MSCs after CRISPRko of *H2AZ1* by von Kossa staining, Toluidine Blue O staining and Oil Red O staining, respectively. Scale bars, 100 μm (M and N), and 20 μm (O). *n* = 3 (M and O), and 6 (N) biological replicates. Data are presented as the means ± SEMs. Two-tailed unpaired Student's t test was performed.

(P) Copy number variation (CNV) analysis in RS hMSCs after CRISPRko of *H2AZ1*.

(Q) Immunofluorescence staining of EdU in RS hMSCs after CRISPRko of *H2AZ1*. Scale bars, 10 μm. *n* = 3 biological replicates. Data are presented as the means ± SEMs. Two-tailed unpaired Student's t test was performed. The white arrowheads indicate the EdU-positive cells.

(R) Immunofluorescence staining of LAP2 in RS hMSCs after CRISPRko of *H2AZ1*. Scale bars, 10 μm. *n* = 300 cells from three biological replicates. Data are presented as the means ± SEMs. Two-tailed unpaired Student's t test was performed.

(S) Heatmap showing the relative mRNA level for indicated genes in RS hMSCs after CRISPRko of *H2AZ1*.

(T) ELISA analysis for the interleukin-6 (IL-6) secretion in RS hMSCs after CRISPRko of *H2AZ1*. Data are presented as the means ± SEMs. *n* = 3 biological replicates. Two-tailed unpaired Student's t test was performed.

(U) Flow cytometric analysis of ROS levels by H2DCFDA staining in RS hMSCs after CRISPRko of *H2AZ1*. Data are presented as the means ± SEM. *n* = 3 biological replicates. Two-tailed unpaired Student's t test was performed.

(V) Heatmap showing sample repeatability of RNA–seq in RS hMSCs after CRISPRko of *H2AZ1*. The color key from blue to white indicates Euclidean distance.

(W) Volcano plot showing the upregulated (red) and downregulated (blue) DEGs in RS hMSCs after CRISPRko of *H2AZ1*.

Figure S2. CRISPRko of *H2AZ1* attenuates senescence in WS and HGPS hMSCs.

(A, G) Western blot analysis of H2AZ1 in WS (A) or HGPS (G) hMSCs after CRISPRko of *H2AZ1*. GAPDH was used as the loading control.

(B, H) SA-β-gal staining of WS (B) or HGPS (H) hMSCs after CRISPRko of *H2AZ1*. Scale bars, 100 μm. *n* = 3 biological replicates. Data are presented as the means ± SEMs. Two-tailed unpaired Student's t test was performed.

(C, I) Clonal expansion analysis of WS (C) or HGPS (I) hMSCs after CRISPRko of *H2AZ1*. *n* = 3 biological replicates. Data are presented as the means ± SEMs. Two-tailed unpaired Student's t test was performed.

(D, J) Immunofluorescence staining of Ki67 in WS (D) or HGPS (J) hMSCs after CRISPRko of *H2AZ1*. Scale bars, 10 μm. *n* = 3 biological replicates. Data are presented as the means ± SEMs. Two-tailed unpaired Student's t test was performed. The white arrowheads indicate the Ki67-positive cells.

(E, K) Heatmap showing the relative mRNA level for the indicated genes in WS (E) or HGPS (K) hMSCs after CRISPRko of *H2AZ1*.

(F, L) Western blot analysis of indicated protein levels in WS (F) or HGPS (L) hMSCs after CRISPRko of *H2AZ1*. GAPDH was used as the loading control. *n* = 3 biological replicates. Quantitative data (right) are presented as the means ± SEMs. Two-tailed unpaired Student's t test was performed.

Figure S3. CRISPRko of *H2AZ1* alleviates UV-, H_2O_2- and oncogene (H-Ras^{V12})-activation-induced cellular senescence.

(A, E, I) Schematic diagram of studies of the role of H2AZ1 in regulating cellular senescence induced by UV (A), H_2O_2 (E) and oncogene (H-Ras^{V12}) transduction (I).

(B, F, J) SA-β-gal staining of *H2AZ1*-CRISPRko hMSCs after treatment with UV (B), H_2O_2 (F) and oncogene (H-Ras^{V12}) transduction (J). Scale bars, 100 μm. *n* = 3 biological replicates. Data are presented as the means ± SEMs. Two-tailed unpaired Student's t test was performed.

(C, G, K) Clonal expansion analysis of *H2AZ1*-CRISPRko hMSCs after treatment with UV (C), H_2O_2 (G) and oncogene (H-Ras^{V12}) transduction (K). *n* = 3 biological replicates. Data are presented as the means ± SEM. Two-tailed unpaired Student's t test was performed.

(D, H, L) Immunofluorescence staining of Ki67 in *H2AZ1*-CRISPRko hMSCs after treatment with UV (D), H_2O_2 (H) and oncogene (H-Ras^{V12}) transduction (L). Scale bars, 20 μm. *n* = 3 biological replicates. Data are presented as the means ± SEM. Two-tailed unpaired Student's t test was performed. White arrows indicate Ki67-positive cells.

Figure S4. Overexpression of H2AZ1 promotes cellular senescence.

(A) Western blot analysis of the indicated proteins level in young hMSCs

transduced with lentiviruses expressing GAL4 or H2AZ1. GAPDH was used as the loading control. *n* = 3 biological replicates. Quantitative data (right) are presented as the means ± SEMs. Two-tailed unpaired Student's t test was performed.

(B) Heatmap showing sample repeatability of RNA–seq in young hMSCs transduced with lentiviruses expressing GAL4 or H2AZ1. The color key from blue to white indicates Euclidean distance.

(C) Volcano plot showing the upregulated (red) and downregulated (blue) DEGs in young hMSCs transduced with lentiviruses expressing H2AZ1 compared to GAL4.

Figure S5. H2AZ1 *trans*-represses *PTN* enhancers.

(A) Heatmap showing sample repeatability of H2AZ1 ChIP–seq in RS hMSCs after CRISPRko of *H2AZ1*. The color key from blue to white indicates Euclidean distance.

(B, C) Bar plots showing the peak number of H2AZ1 (B) and the percentage of H2AZ1 peak coverage across the genome (C) in RS hMSCs after CRISPRko of *H2AZ1*.

(D) Metaplot showing the H2AZ1 ChIP–seq signals at H2AZ1-binding regions in RS hMSCs after CRISPRko of *H2AZ1*.

(E) Scatter plot showing the differential H2AZ1 peaks based on H2AZ1 ChIP–seq in RS hMSCs after CRISPRko of *H2AZ1*. The red dots and blue dots indicate H2AZ1-gained sites and H2AZ1-lost sites, respectively.

(F) Pie plots showing the genomic elements anchored by H2AZ1 in RS hMSCs after CRISPRko of *H2AZ1*.

(G) Venn diagram showing the H2AZ1-regulating genes.

(H) Western blot analysis of PTN in RS hMSCs (EP; LP). GAPDH was used as the loading control. *n* = 3 biological replicates. Quantitative data (right) are presented as the means ± SEMs. Two-tailed unpaired Student's t test was performed.

(I) Enhancers of *PTN* characterized by loss of H2AZ1 signals in RS hMSCs after CRISPRko of *H2AZ1*.

(J, K) RT–qPCR analysis of *PTN* in RS hMSCs after CRISPRko of *H2AZ1* (J) and young hMSCs transduced with lentiviruses expressing GAL4 or H2AZ1 (K). *n* = 3 biological replicates. Data are presented as the means ± SEMs. Two-tailed unpaired Student's t test was performed.

(L) Snapshot showing the RNA–seq signals of *PTN* that upregulated in RS

hMSCs after CRISPRko of *H2AZ1* but downregulated in young hMSCs transduced with lentiviruses expressing H2AZ1.

(M) ChIP–qPCR analysis of the enrichment of H3K27ac on the *PTN* enhancers (E1-E5) in RS hMSCs after CRISPRko of *H2AZ1*. *n* = 3 biological replicates. Data are presented as the means ± SEMs. Two-tailed unpaired Student's t test was performed.

Supplementary Table Legends

Table S1. DEGs identified by RNA-seq analysis in RS hMSCs with H2AZ1 knockout and young hMSCs with H2AZ1 overexpression.

Table S2. *PTN* enhancer sequences bound by H2AZ1.

Table S3. Information of sgRNAs, primers and antibodies.

References

Bi, S., Liu, Z., Wu, Z., Wang, Z., Liu, X., Wang, S., Ren, J., Yao, Y., Zhang, W., and Song, M. (2020). SIRT7 antagonizes human stem cell aging as a heterochromatin stabilizer. Protein & cell 11, 483-504.

Jing, Y., Jiang, X., Ji, Q., Wu, Z., Wang, W., Liu, Z., Guillen-Garcia, P., Esteban, C.R., Reddy, P., and Horvath, S. (2023). Genome-wide CRISPR activation screening in senescent cells reveals SOX5 as a driver and therapeutic target of rejuvenation. Cell Stem Cell 30, 1452-1471. e1410.

Joung, J., Konermann, S., Gootenberg, J.S., Abudayyeh, O.O., Platt, R.J., Brigham, M.D., Sanjana, N.E., and Zhang, F. (2017). Genome-scale CRISPR-Cas9 knockout and transcriptional activation screening. Nature protocols 12, 828-863.

Kim, D., Langmead, B., and Salzberg, S.L. (2015). HISAT: a fast spliced aligner with low memory requirements. Nature methods 12, 357-360.

Langmead, B., and Salzberg, S.L. (2012). Fast gapped-read alignment with Bowtie 2. Nature methods 9, 357-359.

Li, H., Handsaker, B., Wysoker, A., Fennell, T., Ruan, J., Homer, N., Marth, G., Abecasis, G., Durbin, R., and Subgroup, G.P.D.P. (2009). The sequence alignment/map format and SAMtools. bioinformatics 25, 2078-2079.

Li, H., Wu, Z., Liu, X., Zhang, S., Ji, Q., Jiang, X., Liu, Z., Wang, S., Qu, J., and Zhang, W. (2020). ALKBH1 deficiency leads to loss of homeostasis in human diploid somatic cells. Protein & cell 11, 688-695.

Li, L.-Z., Yang, K., Jing, Y., Fan, Y., Jiang, X., Wang, S., Liu, G.-H., Qu, J., Ma, S., and Zhang, W. (2023). CRISPR-based screening identifies XPO7 as a positive regulator of senescence. Protein & Cell, pwad012.

Li, W., Xu, H., Xiao, T., Cong, L., Love, M.I., Zhang, F., Irizarry, R.A., Liu, J.S., Brown, M., and Liu, X.S. (2014). MAGeCK enables robust identification of essential genes from genome-scale CRISPR/Cas9 knockout screens. Genome biology 15, 1-12.

Liao, Y., Smyth, G.K., and Shi, W. (2014). featureCounts: an efficient general purpose program for assigning sequence reads to genomic features. Bioinformatics 30, 923-930.

Liu, G.-H., Qu, J., Suzuki, K., Nivet, E., Li, M., Montserrat, N., Yi, F., Xu, X., Ruiz, S., and Zhang, W. (2012). Progressive degeneration of human neural stem cells caused by pathogenic LRRK2. Nature 491, 603-607.

Love, M.I., Huber, W., and Anders, S. (2014). Moderated estimation of fold change and dispersion for RNA-seq data with DESeq2. Genome biology 15, 1-21.

Ramírez, F., Ryan, D.P., Grüning, B., Bhardwaj, V., Kilpert, F., Richter, A.S., Heyne, S., Dündar, F., and Manke, T. (2016). deepTools2: a next generation web server for deep-sequencing data analysis. Nucleic acids research 44, W160.

Ross-Innes, C.S., Stark, R., Teschendorff, A.E., Holmes, K.A., Ali, H.R., Dunning, M.J., Brown, G.D., Gojis, O., Ellis, I.O., and Green, A.R. (2012). Differential oestrogen receptor binding is associated with clinical outcome in breast cancer. Nature 481, 389-393.

Wang, W., Zheng, Y., Sun, S., Li, W., Song, M., Ji, Q., Wu, Z., Liu, Z., Fan, Y., and Liu, F. (2021). A genome-wide CRISPR-based screen identifies KAT7 as a driver of

cellular senescence. Science translational medicine 13, eabd2655.
Wu, Z., Zhang, W., Song, M., Wang, W., Wei, G., Li, W., Lei, J., Huang, Y., Sang, Y., and Chan, P. (2018). Differential stem cell aging kinetics in Hutchinson-Gilford progeria syndrome and Werner syndrome. Protein & cell 9, 333-350.
Yu, G., Wang, L.-G., and He, Q.-Y. (2015). ChIPseeker: an R/Bioconductor package for ChIP peak annotation, comparison and visualization. Bioinformatics 31, 2382-2383.
Zhang, W., Li, J., Suzuki, K., Qu, J., Wang, P., Zhou, J., Liu, X., Ren, R., Xu, X., and Ocampo, A. (2015). A Werner syndrome stem cell model unveils heterochromatin alterations as a driver of human aging. Science 348, 1160-1163.
Zhao, H., Ji, Q., Wu, Z., Wang, S., Ren, J., Yan, K., Wang, Z., Hu, J., Chu, Q., and Hu, H. (2022). Destabilizing heterochromatin by APOE mediates senescence. Nature Aging 2, 303-316.
Zhou, Y., Zhou, B., Pache, L., Chang, M., Khodabakhshi, A.H., Tanaseichuk, O., Benner, C., and Chanda, S.K. (2019). Metascape provides a biologist-oriented resource for the analysis of systems-level datasets. Nature communications 10, 1523.
