# Supplementary material for: CRISPR-based screening pinpoints H2AZ1 as a driver of senescence in human mesenchymal stem cells
Source: Protein Cell. 2024 Jun 19;16(4):293–9. doi: 10.1093/procel/pwae035 (PMC12082294; doi:10.1093/procel/pwae035)

Figure S1

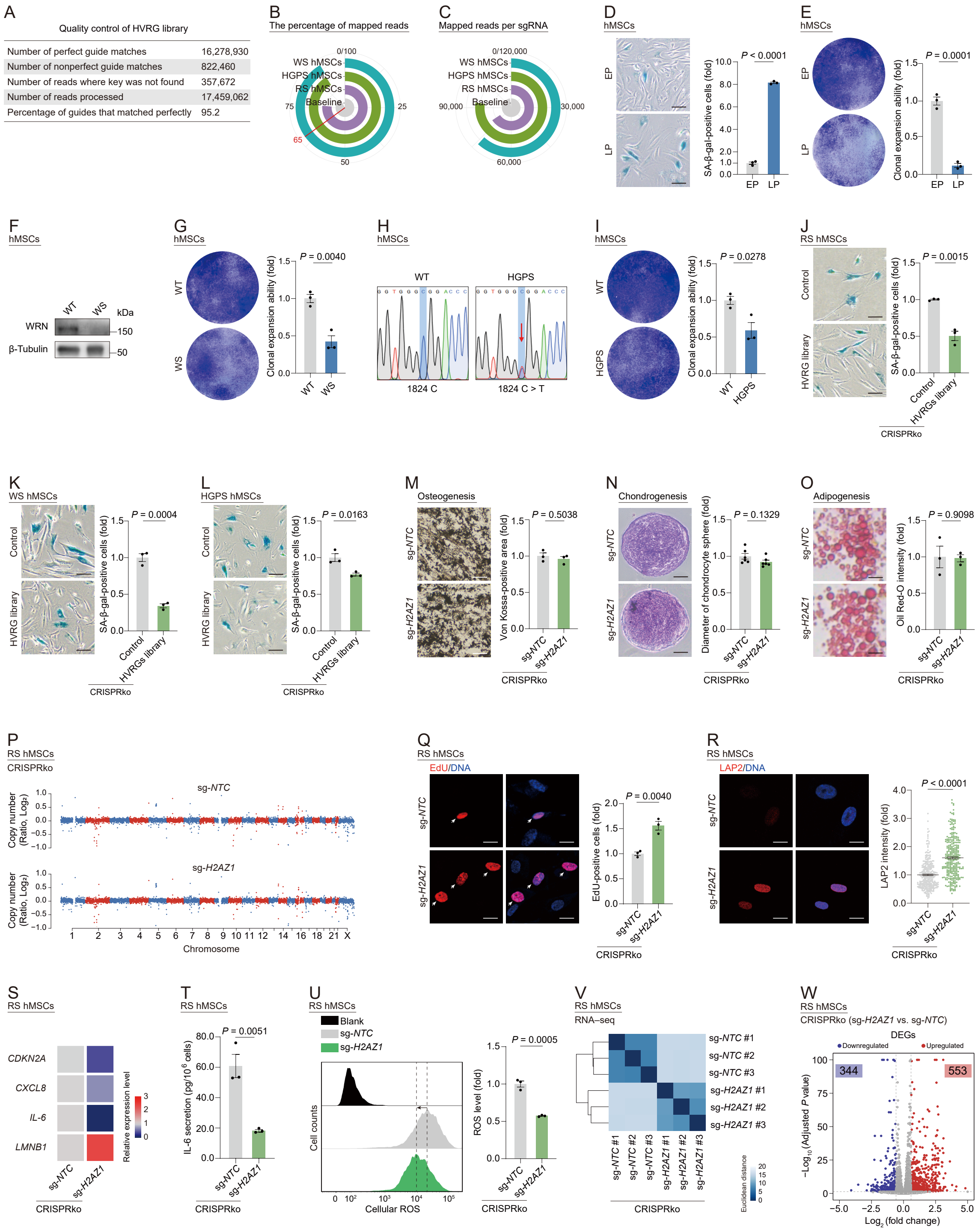

A
Quality control of HVRG library
Number of perfect guide matches 16,278,930
Number of nonperfect guide matches 822,460
Number of reads where key was not found 357,672
Number of reads processed 17,459,062
Percentage of guides that matched perfectly 95.2
B
The percentage of mapped reads
WS hMSCs
HGPS hMSCs
RS hMSCs
Baseline
C
Mapped reads per sgRNA
D
hMSCs
EP
LP
SA-β-gal-positive cells (fold)
P < 0.0001
E
hMSCs
Clonal expansion ability (fold)
P = 0.0001
F
hMSCs
WRN
β-Tubulin
kDa
G
hMSCs
WT
WS
P = 0.0040
H
hMSCs
HGPS
1824 C
1824 C > T
I
P = 0.0278
J
RS hMSCs
Control
HVRG library
HVRGs library
CRISPRko
P = 0.0015
K
WS hMSCs
P = 0.0004
L
HGPS hMSCs
P = 0.0163
M
Osteogenesis
Von Kossa-positive area (fold)
P = 0.5038
sg-NTC
sg-H2AZ1
N
Chondrogenesis
Diameter of chondrocyte sphere (fold)
P = 0.1329
O
Adipogenesis
Oil Red-O intensity (fold)
P = 0.9098
P
Copy number (Ratio, Log2)
Chromosome
Q
EdU/DNA
EdU-positive cells (fold)
R
LAP2/DNA
LAP2 intensity (fold)
S
CDKN2A
CXCL8
IL-6
LMNB1
Relative expression level
T
IL-6 secretion (pg/10^6 cells)
P = 0.0051
U
Blank
Cellular ROS
Cell counts
ROS level (fold)
P = 0.0005
V
RNA–seq
sg-NTC #1
sg-NTC #2
sg-NTC #3
sg-H2AZ1 #1
sg-H2AZ1 #2
sg-H2AZ1 #3
Euclidean distance
W
CRISPRko (sg-H2AZ1 vs. sg-NTC)
DEGs
Downregulated
Upregulated
344
553
−Log10 (Adjusted P value)
Log2 (fold change)

Figure S2

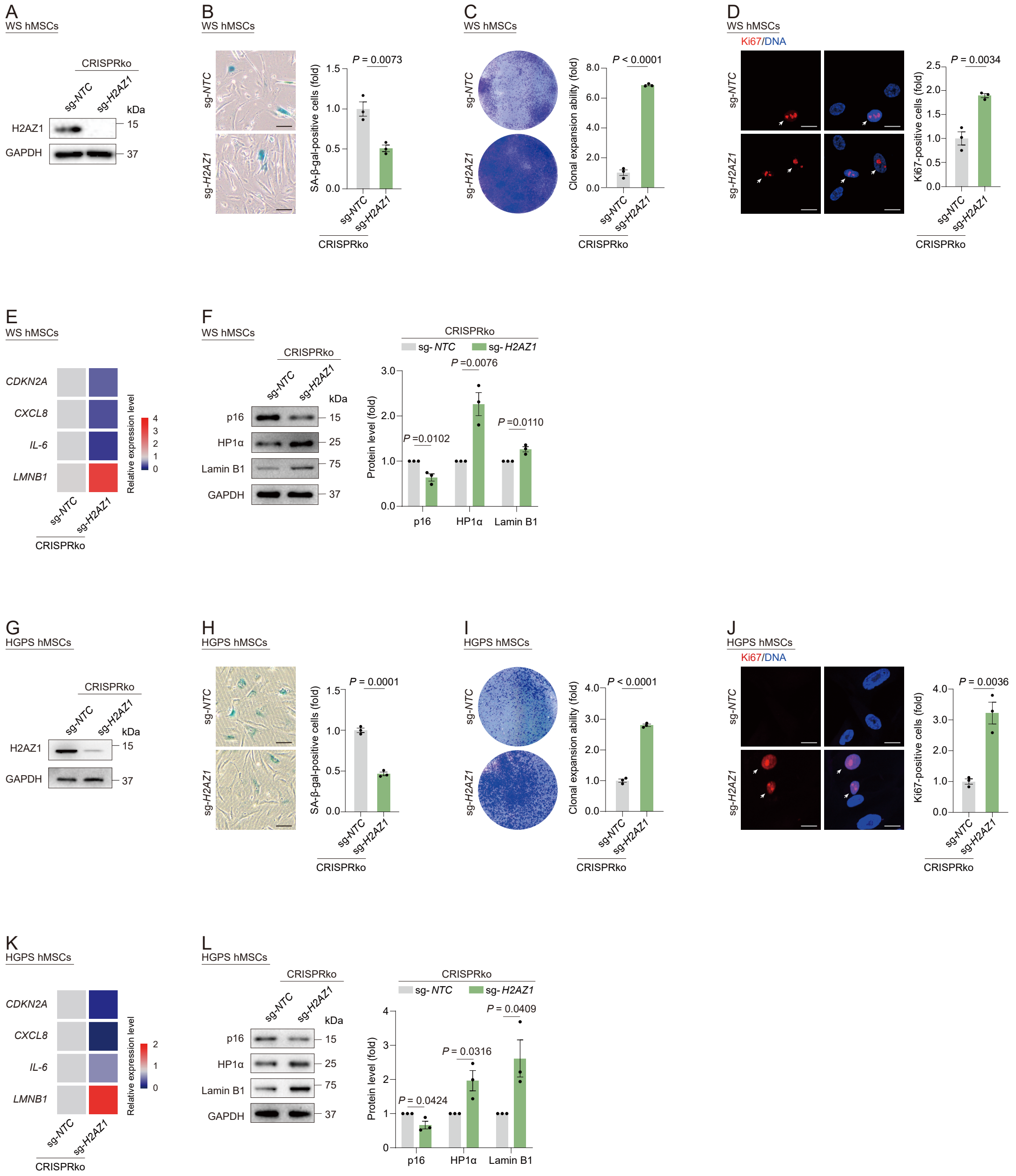

A
WS hMSCs
CRISPRko
sg-NTC
sg-H2AZ1
kDa
H2AZ1
15
GAPDH
37
B
WS hMSCs
sg-NTC
sg-H2AZ1
P = 0.0073
SA-β-gal-positive cells (fold)
CRISPRko
C
WS hMSCs
P < 0.0001
Clonal expansion ability (fold)
D
WS hMSCs
Ki67/DNA
P = 0.0034
Ki67-positive cells (fold)
E
WS hMSCs
CDKN2A
CXCL8
IL-6
LMNB1
Relative expression level
F
WS hMSCs
p16
HP1α
Lamin B1
GAPDH
P =0.0076
P =0.0102
P =0.0110
Protein level (fold)
G
HGPS hMSCs
H
HGPS hMSCs
P = 0.0001
I
HGPS hMSCs
P < 0.0001
J
HGPS hMSCs
P = 0.0036
K
HGPS hMSCs
L
HGPS hMSCs
P = 0.0409
P = 0.0316
P = 0.0424

Figure S3

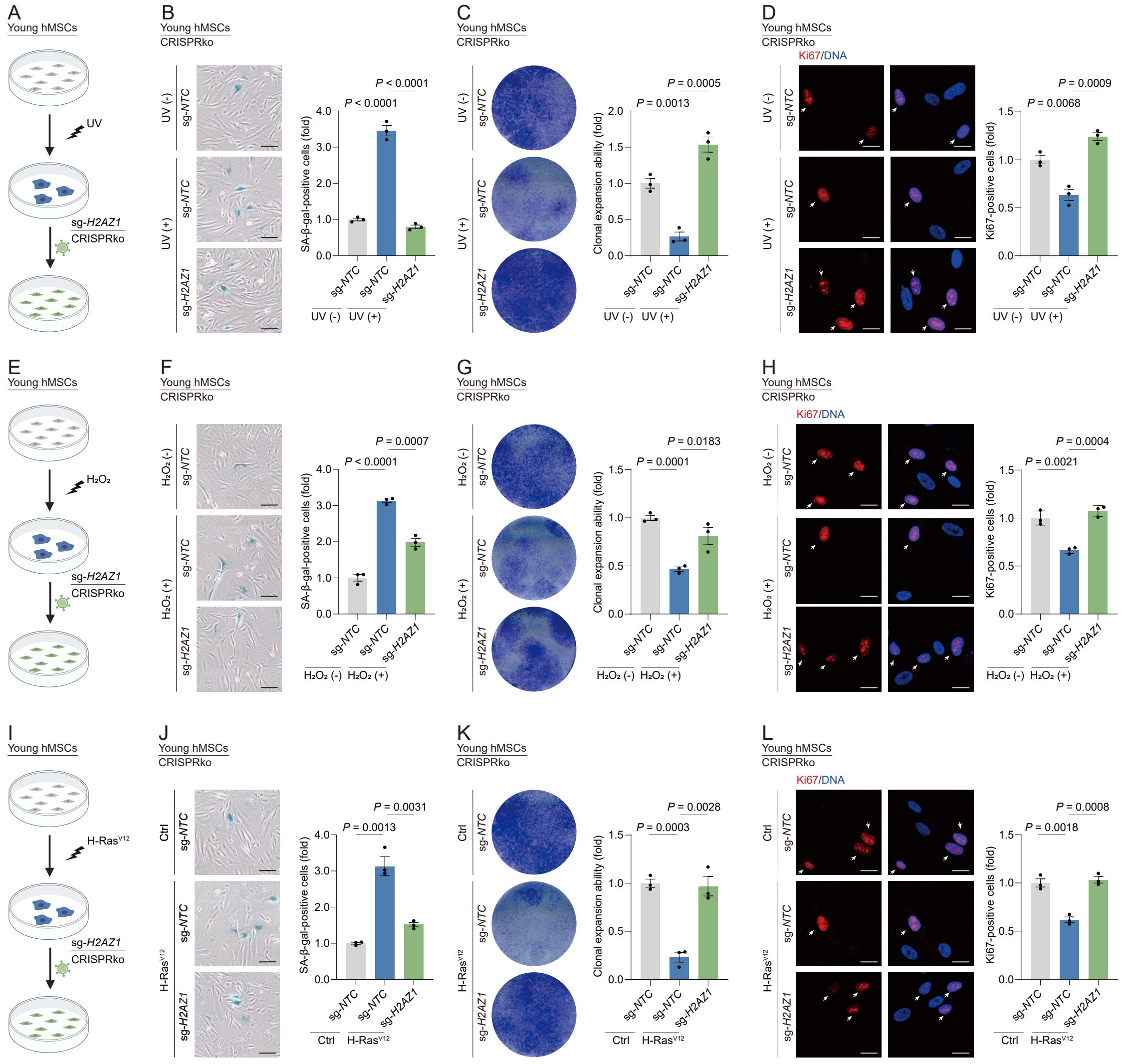
A
Young hMSCs
UV
sg-H2AZ1
CRISPRko
B
Young hMSCs
CRISPRko
UV (-)
UV (+)
sg-NTC
sg-H2AZ1
SA-β-gal-positive cells (fold)
P < 0.0001
P < 0.0001
C
Young hMSCs
CRISPRko
Clonal expansion ability (fold)
P = 0.0005
P = 0.0013
D
Young hMSCs
CRISPRko
Ki67/DNA
Ki67-positive cells (fold)
P = 0.0009
P = 0.0068
E
Young hMSCs
H₂O₂
sg-H2AZ1
CRISPRko
F
Young hMSCs
CRISPRko
H₂O₂ (-)
H₂O₂ (+)
P = 0.0007
P < 0.0001
G
Young hMSCs
CRISPRko
P = 0.0183
P = 0.0001
H
Young hMSCs
CRISPRko
Ki67/DNA
P = 0.0004
P = 0.0021
I
Young hMSCs
H-RasV12
sg-H2AZ1
CRISPRko
J
Young hMSCs
CRISPRko
Ctrl
H-RasV12
P = 0.0031
P = 0.0013
K
Young hMSCs
CRISPRko
P = 0.0028
P = 0.0003
L
Young hMSCs
CRISPRko
Ki67/DNA
P = 0.0008
P = 0.0018

Figure S4

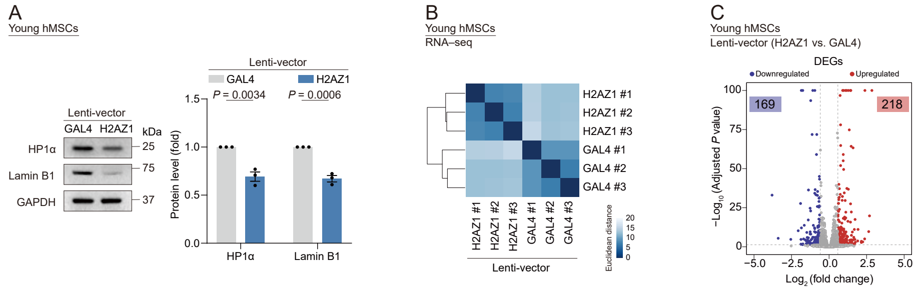

A
Young hMSCs
Lenti-vector
GAL4
H2AZ1
kDa
HP1α
Lamin B1
GAPDH
25
75
37
Lenti-vector
GAL4
H2AZ1
P = 0.0034
P = 0.0006
Protein level (fold)
1.5
1.0
0.5
0.0
HP1α
Lamin B1
B
Young hMSCs
RNA–seq
H2AZ1 #1
H2AZ1 #2
H2AZ1 #3
GAL4 #1
GAL4 #2
GAL4 #3
Lenti-vector
Euclidean distance
20
15
10
5
0
C
Young hMSCs
Lenti-vector (H2AZ1 vs. GAL4)
DEGs
Downregulated
Upregulated
169
218
−Log10 (Adjusted P value)
100
75
50
25
0
−5.0
−2.5
0.0
2.5
5.0
Log2 (fold change)

Figure S5

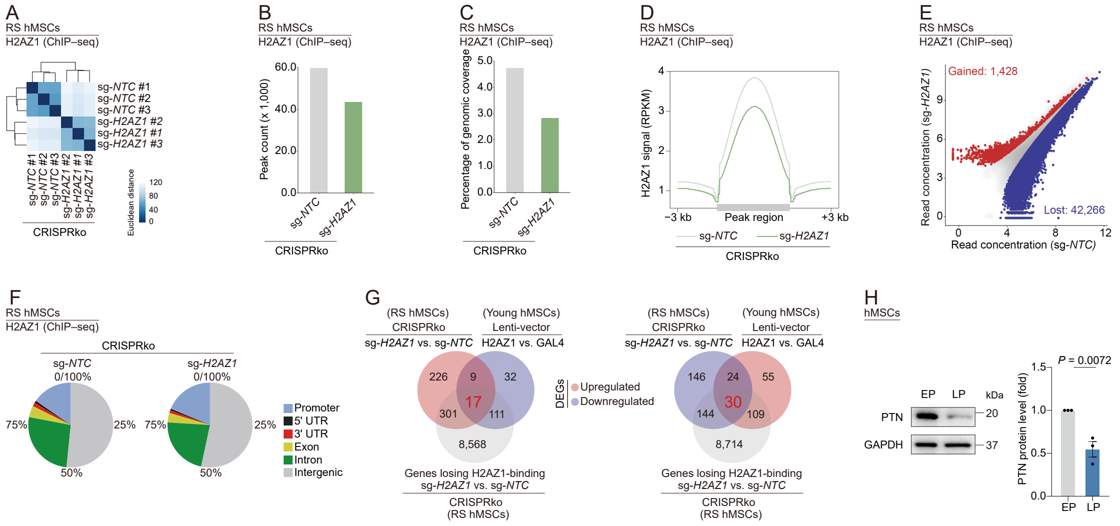

I

PTN enhancer region bound by H2AZ1

	Chr	Start	End	Log_2 (fold change)	FDR
PTN E1	Chr7	137161400	137162099	-1.16	6.84E-03
PTN E2	Chr7	137192300	137196099	-0.66	5.16E-06
PTN E3	Chr7	137221900	137225399	-0.63	3.22E-05
PTN E4	Chr7	137259300	137269299	-0.92	5.07E-04
PTN E5	Chr7	137274300	137278399	-0.46	1.91E-03

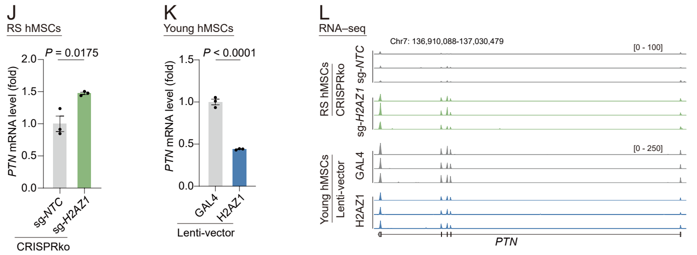

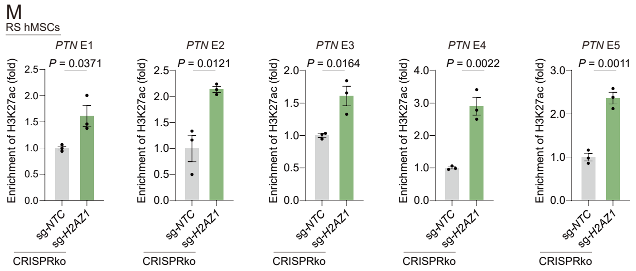

Supplement: pwae035_suppl_Supplementary_Figures_S1-S5 [file pwae035_suppl_supplementary_figures_s1-s5.pdf]
